# Prevalence of high-risk human papillomavirus types in Mexican women with cervical intraepithelial neoplasia and invasive carcinoma

**DOI:** 10.1186/1750-9378-3-3

**Published:** 2008-02-28

**Authors:** Rubén López-Revilla, Luz A Martínez-Contreras, Mireya Sánchez-Garza

**Affiliations:** 1 División de Biología Molecular, Instituto Potosino de Investigación Científica y Tecnológica, Camino a la Presa San José 2055, 78216 San Luis Potosí, S.L.P., México

## Abstract

**Background:**

Prevalence of high risk (HR) human papillomavirus (HPV) types in the states of San Luis Potosí (SLP) and Guanajuato (Gto), Mexico, was determined by restriction fragment length-polymorphism (RFLP) analysis on the E6 ~250 bp (E6-250) HR-HPV products amplified from cervical scrapings of 442 women with cervical intraepithelial neoplasia and invasive carcinoma (280 from SLP and 192 from Gto). Fresh cervical scrapings for HPV detection and typing were obtained from all of them and cytological and/or histological diagnoses were performed on 383.

**Results:**

Low grade intraepithelial squamous lesions (LSIL) were diagnosed in 280 cases (73.1%), high grade intraepithelial squamous lesions (HSIL) in 64 cases (16.7%) and invasive carcinoma in 39 cases (10.2%). In the 437 cervical scrapings containing amplifiable DNA, only four (0.9%) were not infected by HPV, whereas 402 (92.0%) were infected HR-HPV and 31 (7.1%) by low-risk HPV. RFLP analysis of the amplifiable samples identified infections by one HR-HPV type in 71.4%, by two types in 25.9% and by three types in 2.7%. The overall prevalence of HR-HPV types was, in descending order: 16 (53.4%) > 31 (15.6%) > 18 (8.9%) > 35 (5.6) > 52 (5.4%) > 33 (1.2%) > 58 (0.7%) = unidentified types (0.7%); in double infections (type 58 absent in Gto) it was 16 (88.5%) > 31 (57.7%) > 35 (19.2%) > 18 (16.3%) = 52 (16.3%) > 33 (2.8%) = 58 (2.8%) > unidentified types (1.0%); in triple infections (types 33 and 58 absent in both states) it was 16 (100.0%) > 35 (54.5%) > 31 (45.5%) = 52 (45.5%) > 18 (27.3%). Overall frequency of cervical lesions was LSIL (73.1%) > HSIL (16.7%) > invasive cancer (10.2%). The ratio of single to multiple infections was inversely proportional to the severity of the lesions: 2.46 for LSIL, 2.37 for HSIL and 2.15 for invasive cancer. The frequency of HR-HPV types in HSIL and invasive cancer lesions was 16 (55.0%) > 31 (18.6%) > 35 (7.9%) > 52 (7.1%) > 18 (4.3%) > unidentified types (3.6%) > 33 (2.9%) > 58 (0.7%).

**Conclusion:**

Ninety percent of the women included in this study were infected by HR-HPV, with a prevalence 1.14 higher in Gto. All seven HR-HPV types identifiable with the PCR-RFLP method used circulate in SLP and Gto, and were diagnosed in 99.3% of the cases. Seventy-one percent of HR-HPV infections were due to a single type, 25.9% were double and 2.7% were triple. Overall frequency of lesions was LSIL (73.1%) > HSIL (16.7%) > invasive cancer (10.2%), and the ratio of single to multiple infections was inversely proportional to severity of the lesions: 2.46 for LSIL, 2.37 for HSIL and 2.15 for invasive cancer. The frequency of HR-HPV types found in HSIL and invasive cancer was 16 (55.0%) > 31 (18.6%) > 35 (7.9%) > 52 (7.1%) > 18 (4.3%) > unidentified types (3.6%) > 33 (2.9%) > 58 (0.7%). Since the three predominant types (16, 31 and 18) cause 77.9% of the HR-HPV infections and immunization against type 16 prevents type 31 infections, in this region the efficacy of the prophylactic vaccine against types 16 and 18 would be close to 80%.

## Background

Cervical cancer (CC) is the second cause of death by cancer among women in the world and the first in most developing countries [1,2]. In the year 2000, 190,000 deaths and 80% of the 500,000 new CC cases occurred in the developing world, and Latin American countries were among those with the highest incidence rates, together with countries from Sub-Saharan Africa, South and South East Asia [3]. The risk of developing CC increases with early start of sexual activity, number of sexual partners, prolonged use of oral contraceptives and smoking [4]. In Mexico it is associated with poverty related factors such as low schooling, unemployment, residence in rural areas and lack of access to health services [5-7].

zur Hausen [8] proposed and later demonstrated [9] that human papillomaviruses (HPV) are the infectious agents responsible of the neoplastic transformation of the cervical epithelium. This hypothesis was validated by finding HPV genome sequences in 99.7% of invasive CC cases [10]. It is currently accepted that HPV is the most common sexually transmitted pathogen [11] and that infection of the cervix by 'high risk' (HR) HPV types is a necessary factor for CC development [10,12]. Low risk (LR) HPV types are those usually found in warts and benign lesions whereas HR types are those found in invasive CC [13].

There are 11 major LR-HPV types (designated as 6, 11, 40, 42, 43, 44, 54, 61, 70, 72, 81) and 15 major HR-HPV types (designated as 16, 18, 31, 33, 35, 39, 45, 51, 52, 56, 58, 59, 68, 73, 82) [14]. The two most frequently associated to malignancies are HR-HPV types 16 and 18. The first is responsible for nearly 50% and the second for nearly 20% of all invasive CC cases in the world [10,15]. Several HR-HPV variants with higher oncogenic potential are more prevalent in developing countries, where they appear to contribute to the higher incidence and mortality rates. In Mexico the probability of developing CC by the Asian-American variant of HPV type 16 is several times higher than by the European variant, and around 25% of invasive CC cases are attributed to the AA variant [16].

During the year 2000 the incidence of CC in Mexico was 40.5 cases per 100,000 women over 25 years of age and the mortality rate was 17.1 [3], which means 12 deaths per day. In the Mexican states of San Luis Potosí (SLP) and Guanajuato (Gto) the mortality rates for the same year were respectively 15.5 and 21.7 [17]. Lazcano-Ponce et al. [18] found a HPV prevalence of 16.7% in Mexican women below 25 years of age which decreased to 3.7% in those of 25–44 years and increased to 23% in those above 65 years, and that HR-HPV types are more prevalent in all age groups whereas LR-HPV types are less prevalent in women under 25 years and increase with age.

In the present work we determined the prevalence of the HR-HPV types circulating in the neighboring states of San Luis Potosí and Guanajuato by restriction fragment length polymorphism (RFLP) analysis with the method of Fujinaga et al. [19], in women with precancerous and cancerous lesions of the cervix that had been diagnosed by cytological analysis; in two thirds of them biopsies or surgical specimens were obtained and the microscopic diagnosis confirmed.

## Results

### Study population

The 442 women enrolled in this study ranged from 16 to 78 years of age. Fresh cervical scrapings for HPV detection and typing were obtained from all of them whereas cytological and/or histological diagnoses were performed on 383. Low grade intraepithelial squamous lesions (LSIL) were diagnosed in 280 cases (73.1%), high grade intraepithelial squamous lesions (HSIL) in 64 cases (16.7%) and invasive carcinoma in 39 cases (10.2%).

### Algorithm for HPV DNA amplification of cervical scrapings

Amplification of the HR-HPV E6-250 product by direct and nested PCR was compared. Direct PCR was performed with primer set pU 1M/2R whereas nested PCR was performed in two successive reactions: in the first one ('direct') the E6-650 segment was amplified using primer set LCRS/E7AS, whereas in the second one ('nested') a portion of the first reaction mixture (presumed to contain the E6-650 product) was used as template for the internal primers (pU 1M/2R) to generate E6-250.

Amplification of 35 random samples showed that 11 (31.4%) were positive by direct PCR and 24 (68.6%) by nested PCR. Since E6-250 detection was 2.2 times more sensitive with nested PCR, this modality was chosen to analyze all problem samples.

**Figure 1:**
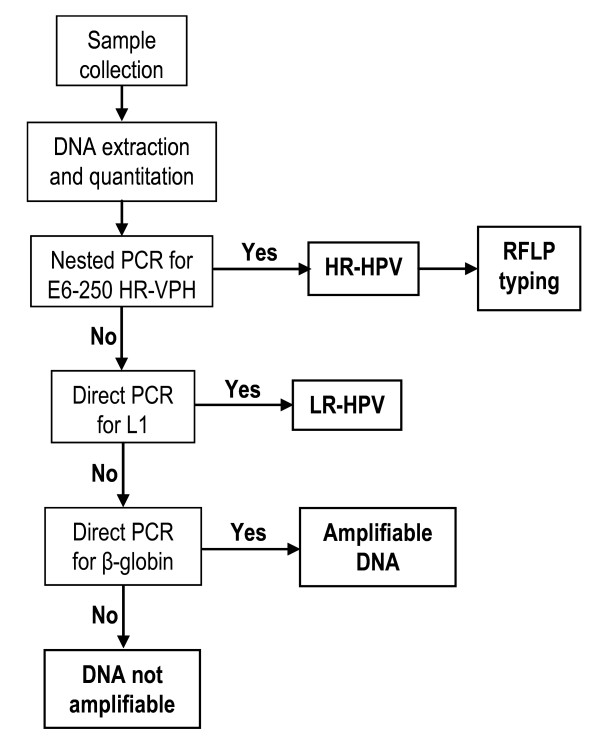
**Algorithm for high-risk HPV detection in cervical scrapings from women with dysplastic and neoplastic lesions**. It checks first for the presence of HPV DNA through nested PCR (expected to be positive in most samples) and then for the amplificability of β-globin DNA in HPV-negative samples (expected to be rare).

**Figure 2:**
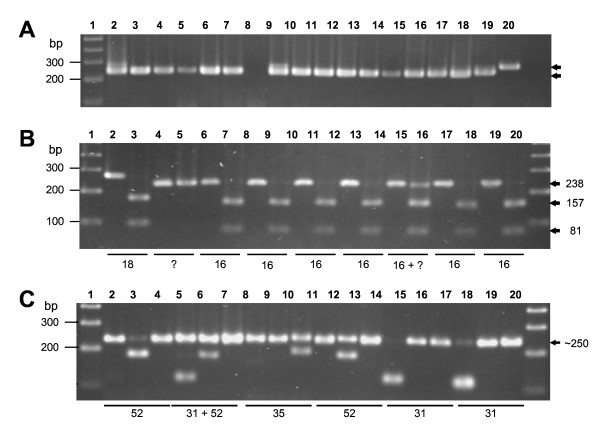
**HR-HPV DNA detection and typing**. Examples of agarose gels used for detection of the E-250 products amplified by nested PCR, for the first stage of HR-HPV typing with *Ava *II, and for the second stage with *Rsa *I, *Bgl *II, *Ava *I and *Acc *I endonucleases. Numbers to the left indicate the size of the DNA markers; arrows to the right indicate the size of the products amplified or the restriction fragments obtained. (**A**) HR-HPV DNA detection. Lane 1, 100 bp ladder. Lanes 2–18, DNA from different patients. Lane 19, Positive control (HeLa cell DNA). Lane 20, Negative control (no DNA). The expected E6-250 band appeared in the positive control and all problem samples except that of lane 8; Note the doublets in lanes 2 and 9 suggestive of double infection. (**B**) Identification of HPV types 16 and 18 by restriction with *Ava *II. Lanes 1 and 20, 100 bp ladder. Lanes 2 and 3, Positive control: E6-250 product from HeLa cell DNA intact (lane 2) and treated with *Ava *II (lane 3). Lanes 4–19: neighboring lanes containing E6-250 products either intact or treated with *Ava *II. Lanes 4 and 5, Patient 322. Lanes 6 and 7, Patient 323. Lanes 8 and 9, Patient 324. Lanes 10 and 11, Patient 325. Lanes 12 and 13, Patient 326. Lanes 14 and 15, Patient 328. Lanes 16 and 17, Patient 329. Lanes 18 and 19, Patient 306. Note the slightly larger size of HPV18 product and fragments, as well as total and partial resistance to *Ava *II by samples from patients 322 (lane 5) and 328 (lane 15). (**C**) Identification of HPV types 31, 52 and 35. Groups of three neighboring lanes contained E6-250 products in mixtures incubated separately with *Rsa *I, *Bgl *II and *Ava *I endonucleases from each sample. Lanes 1 and 20, 100 bp ladder. Lanes 2–4, Patient 191. Lanes 5–7, Patient 197. Lanes 8–10, Patient 203. Lanes 11–13, Patient 209. Lanes 14–16, Patient 211. Lanes 17–19, Patient 246. Note that infections by one and two HPV types are clearly distinguished.

**Figure 3:**
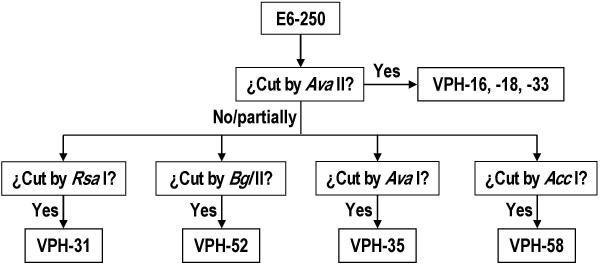
**Algorithm for HR-HPV typing**. The PCR-RFLP method used identifies seven HR-HPV types by the size of the restriction fragments of the E6-250 products generated by specific cuts with five enzymes. *Ava *II is used first because it helps recognize types 16, 18 and 33 (16 and 18 are known to be the most prevalent globally). PCR products not restricted by *Ava *II are incubated separately with each of the four remaining enzymes to identify the other five HR-HPV types.

To determine the presence of HPV DNA in cervical scrapings the algorithm shown in Fig. 1 was used. Each sample is first subjected to nested PCR to successively amplify the E6-650 and E6-250 products. If the second one is visible, the sample is considered positive for HR-HPV (examples in Fig. 2A) and typified by RFLP (examples in Fig. 2B and 3C). If the second product is not observed, the sample could be either positive, inadequate or negative. To define if it is positive it is subjected to PCR with the primer set MY 09/11; if L1-450 is observed, the sample is considered positive for low risk HPV and if L1-450 is not visible, the sample may be negative or of inadequate quality and subjected to PCR with the β-globin primer set. If the β-globin product is observed the sample is considered to be amplifiable but HPV-negative; if it is not visible, the sample is considered inadequate (i.e., not amplifiable) and undefined in relation to HPV infection.

In the analysis of samples from women with cervical dysplasia and neoplasia, as is the case of this study, this algorithm saves labor, time and reagents because it first verifies the presence of HPV through nested PCR (expected to be positive in most samples) and then the amplificability of the HPV-negative samples (expected to be rare) by performing a PCR assay for a single copy housekeeping gene such as that of β-globin.

Using the algorithm for HPV detection (Fig. 1) the 437 cervical scrapings with amplifiable DNA were analyzed. HPV infection was demonstrated in 433 samples (99.1%) of which 402 (92.0%) corresponded to high risk viral types (Table 1).

**Table 1:** Low risk- and high risk-HPV DNA detected in amplifiable samples

Sample type	SLP		Gto		Overall	
	
	**n**	**%**	**n**	**%**	**n**	**%**
HR-HPV (*E6+*)	212	86.5	190	99.0	402	92.0
LR-HPV (*E6*-*, L1+*)	30	12.2	1	0.5	31	7.1
HPV negative (*E6*-*, L1*-*, β-globin+*)	3	1.2	1	0.5	4	0.9

**Total**	**245**	**100.0**	**192**	**100.0**	**437**	**100.0**

HR-HPV, positive for E6-250 product of high risk HPV.

LR-HPV, low-risk HPV (negative for E6-250; positive for L1-450).

### Algorithm for HR-HPV typing

The sizes of E6-250 products and their corresponding restriction fragments for the seven HR-HPV types that can be identified with the PCR-RFLP method used appear in Table 2.

**Table 2:** Size of the E6-250 products and restriction fragments of the seven HR-HPV types identified^a^

Enzyme	HR-HPV type						
	
	16	18	31	33	35	52	58
Total length (bp)	238	268	232	244	232	231	244

*Ava *II	157/81	172/96	NC	136/108	NC	NC	NC
*Rsa *I	NC	NC	117/115	NC	NC	NC	NC
*Ava *I	NC	NC	NC	NC	186/46	NC	NC
*Bgl *II	NC	NC	NC	NC	NC	176/55	NC
*Acc *I	NC	NC	NC	NC	NC	NC	126/118

^a^Taken from Hwang [26].

NC, not cut.

To identify HR-HPV types the algorithm of Fig. 3 was used. The E6-250 product from each HR-HPV type has a specific size and a single restriction site for each of the five enzymes used, which generate two fragments of specific sizes allowing the identification of seven of the most frequent HR-HPV types. *Ava *II, the first enzyme used on each HR-HPV positive sample, identifies types 16, 18 and 33 (see examples in Fig. 2A). If *Ava *II does not digest the E6-250 product or does it only partially, sample aliquots are simultaneously incubated with each of the four remaining enzymes to identify types 31, 52, 35 and 58 (see examples in Fig. 2C).

### HR-HPV types circulating in the region

In 402 of the 435 samples with HPV infection, one to three of the seven identifiable HR-HPV types were detected.

The overall prevalence of HR-HPV types was, in descending order: 16 (53.4%) > 31 (15.6%) > 18 (8.9%) > 35 (5.6) > 52 (5.4%) > 33 (1.2%) > 58 (0.7%) = unidentified types (0.7%). Types 16, 31 and 18 therefore accounted for 77.9% of all the infections whereas the other four types accounted for only 12.9% of them. HPV type 16 predominated slightly in SLP (53.3%) over Gto (52.9%), type 31 in Gto (22.3%) over SLP (10.1%), and type 18 was equally prevalent in SLP and Gto (8.8%) (Table 3).

**Table 3:** Prevalence of HR-HPV types

HR-HPV type	SLP		Gto		Overall	
	
	n	%	n	%	n	%
16	169	53.3	138	52.9	307	53.4
31	32	10.1	58	22.3	90	15.6
18	28	8.8	23	8.8	51	8.9
35	15	4.7	17	6.5	32	5.6
52	16	5.1	15	5.7	31	5.4
33	3	0.9	4	1.6	7	1.2
58	4	1.3	0	0.0	4	0.7
NI	1	0.3	3	1.2	4	0.7

**Total**	**268**	**84.5**	**258**	**99.0**	**526**	**91.4**

NI, type not identified.

### HR-HPV types in single and multiple infections

The overall prevalence of infections by a single HR-HPV type was 71.4%, i.e., 2.5 times higher than that of infections by two and three types (Table 4).

**Table 4:** Prevalence of single and multiple HR-HPV infections

Infections	SLP		Gto		Overall	
	
	n	%	n	%	n	%
Single	159	75.0	128	67.4	287	71.4
Double	48	22.6	56	29.5	104	25.9
Triple	5	2.4	6	3.2	11	2.7

**Total**	**212**	**100.0**	**190**	**100.0**	**402**	**100.0**

The prevalence of infections by a single HR-HPV type was slightly higher in SLP (75.0%) than in Gto (67.4%), whereas the overall prevalence of infections by two and three HR-HPV types was respectively 25.9% and 2.7%, of which 22.6% and 2.4% corresponded to SLP and 29.5% and 3.2% to Gto (Table 4).

The HR-HPV types of single, double and triple infections as well as the combinations of viral types in multiple infections found in all samples and per state are shown in Table 5.

**Table 5:** Prevalence of HR-HPV types in single, double and triple infections

Infections	HR-HPV ypes	SLP		Gto		Overall	
		
		n	%	n	%	n	%
**Single**	16	123	77.4	81	63.3	204	72.6
	18	15	9.4	16	12.5	31	10.2
	31	13	8.2	17	13.3	30	9.9
	52	4	2.5	6	4.7	10	3.3
	33	2	1.3	2	1.6	4	1.3
	35	1	0.6	3	2.3	4	1.3
	58	1	0.6	0	0.0	1	0.3
	NI	0	0.0	3	2.3	3	1.0
	
	**Sum**	**159**	**100.0**	**128**	**100.0**	**287**	**100.0**

**Double**	16/18	7	14.6	4	7.1	11	10.6
	16/31	14	29.2	35	63.0	49	47.1
	16/33	1	2.1	1	1.8	2	1.9
	16/35	7	14.6	6	11.0	13	12.5
	16/52	9	18.8	5	8.9	14	13.5
	16/58	3	6.3	0	0.0	3	2.9
	18/31	1	2.1	1	1.8	2	1.9
	18/35	2	4.2	0	0.0	2	1.9
	18/52	1	2.1	0	0.0	1	1.0
	18/NI	1	2.1	0	0.0	1	1.0
	31/33	0	0.0	1	1.8	1	1.0
	31/35	1	2.1	1	1.8	2	1.9
	35/52	1	2.1	2	3.6	3	2.9
	
	**Sum**	**48**	**100.0**	**56**	**100.0**	**104**	**100.0**

**Triple**	16/31/52	2	40.0	1	17.0	3	27.3
	16/18/35	1	20.0	2	33.0	3	27.3
	16/31/35	1	20.0	2	33.0	3	27.3
	16/35/52	1	20.0	1	17.0	2	18.2
	
	**Sum**	**5**	**100.0**	**6**	**100.0**	**11**	**100.0**

**Total**		**212**	**52.7**	**190**	**47.3**	**402**	**100.0**

NI, type not identified.

#### Single infections

HPV type 16 was the most prevalent (72.6%) followed by types 18 (10.2%) and 31 (9.9%) and then by types 52 (3.3%), 33 (1.3%) = 35 (1.3%), and 58 (0.3%) (Table 5).

The descending order of frequency of the viral types in single infections was clearly different in each state. In SLP the type 16 (77.4%) predominated, followed by types 18 (9.4%), 31 (8.2%), 52 (2.5%), 33 (1.3%), 35 (0.6%) = 58 (0.6%). In Gto the prevalence of type 16 was lower (63.3%) and type 31 was second (13.3%), followed by types 18 (12.5%), 52 (4.7%), 35 (2.3%) and 33 (1.6%) (Table 5).

#### Multiple infections

The overall prevalence of HPV type 16 increased in double infections and reached its maximum possible value (100%) in triple infections (Table 6). The overall prevalence of HPV type 18 also increased in double infections and was accompanied by increases of types 31, 52 and 35 and the last two types were even higher in triple infections (Table 6). In double and triple infections of SLP the predominant types were 16, 31, 18, 35 and 52 whereas in Gto types 16 and 31 predominated in double infections and types 16 and 31 in the triple ones (Table 6).

**Table 6:** Prevalence (%) of HR-HPV types in single, double and triple infections

HR-HPV types	SLP			Gto			Overall		
	
	Single	Double	Triple	Single	Double	Triple	Single	Double	Triple
16	77.4	85.4	100.0	36.7	91.1	100.0	71.1	88.5	100.0
18	9.4	25.0	20.0	10.0	8.9	33.3	10.8	16.3	27.3
31	8.2	33.3	40.0	16.7	78.6	50.0	10.5	57.7	45.5
52	2.5	20.8	60.0	16.7	12.5	33.3	3.5	16.3	45.5
33	1.3	2.1	0.0	0.0	3.6	0.0	0.0	2.9	0.0
35	0.6	22.9	60.0	20.0	16.1	50.0	1.4	19.2	54.5
58	0.6	6.3	0.0	0.0	0.0	0.0	0.3	2.9	0.0
NI	0.0	2.1	0.0	0.0	0.0	0.0	1.0	1.0	0.0

NI, type not identified.

### Single and multiple infections and HR-HPV types in lesions of increasing severity

The overall frequency of cervical lesions was, in decreasing order, LSIL (73.1%) > HSIL (16.7%) > invasive cancer (10.2%) (Table 7). The ratio of single to multiple infections was inversely proportional to the severity of the lesions: 2.46 for LSIL, 2.37 for HSIL and 2.15 for invasive cancer (Table 8).

**Table 7:** HR-HPV types of single and multiple infections in lesions of increasing severity

Infections	HR-HPV types	LSIL		HSIL		Cancer		Overall	
		
		n	%	n	%	n	%	n	%
**Single**	16	149	38.9	37	9.7	17	4.4	203	53.0
	18	21	5.5	0	0.0	1	0.3	22	5.7
	31	17	4.4	5	1.3	4	1.0	26	6.8
	33	0	0.0	1	0.3	2	0.5	3	0.8
	35	4	1.0	1	0.3	1	0.3	6	1.6
	52	7	1.8	1	0.3	1	0.3	9	2.3
	58	1	0.3	0	0.0	0	0.0	1	0.3
	
	**Sum**	**199**	**52.0**	**45**	**11.7**	**26**	**6.8**	**270**	**70.5**

**Double**	16/18	9	2.3	4	1.0	0	0.0	13	3.4
	16/31	36	9.4	4	1.0	9	2.3	49	12.8
	16/33	1	0.3	0	0.0	0	0.0	1	0.3
	16/35	10	2.6	2	0.5	1	0.3	13	3.4
	16/52	8	2.1	3	0.8	1	0.3	12	3.1
	16/58	2	0.5	1	0.3	0	0.0	3	0.8
	18/35	3	0.8	0	0.0	1	0.3	4	1.0
	18/NI	1	0.3	0	0.0	0	0.0	1	0.3
	31/33	0	0.0	1	0.3	0	0.0	1	0.3
	31/35	2	0.5	0	0.0	0	0.0	2	0.5
	31/52	1	0.3	1	0.3	0	0.0	2	0.5
	35/52	0	0.0	1	0.3	0	0.0	1	0.3
	
	**Sum**	**73**	**19.1**	**17**	**4.4**	**12**	**3.1**	**102**	**26.6**

**Triple**	16/18/35	1	0.3	1	0.3	0	0.0	2	0.5
	16/31/35	2	0.5	1	0.3	1	0.3	4	1.0
	16/31/52	2	0.5	0	0.0	0	0.0	2	0.5
	16/35/52	2	0.5	0	0.0	0	0.0	2	0.5
	18/31/52	1	0.3	0	0.0	0	0.0	1	0.3
	
	**Sum**	**8**	**2.1**	**2**	**0.5**	**1**	**0.3**	**11**	**2.9**

	**Total**	**280**	**73.1**	**64**	**16.7**	**39**	**10.2**	**383**	**100.0**

**Table 8:** Frequency of single and multiple HR-HPV infections in lesions of increasing severity

Lesion	Type of infection						S/M ratio
		
	Single (S)		Multiple (M)		Overall		
		
	n	%	n	%	n	%	
LSIL	199	52.0	81	21.1	280	73.1	2.46
HSIL	45	11.7	19	5.0	64	16.7	2.37
Cancer	26	6.8	13	3.4	39	10.2	2.00

**Total**	**280**	**70.5**	**113**	**29.5**	**383**	**100.0**	**---**

LSIL, low-grade squamous intraepithelial lesion.

HSIL, high-grade squamous intraepithelial lesion.

The frequency of HR-HPV types found in the cervical HSIL and invasive cancer (n = 140) was, in decreasing order, 16 (55.0%) > 31 (18.6%) > 35 (7.9%) > 52 (7.1%) > 18 (4.3%) > unidentified types (3.6%) > 33 (2.9%) > 58 (0.7%) (Table 9).

**Table 9:** HR-HPV types in HSIL and invasive cancer lesions

HR-HPV type	Frequency (%)		O/H ratio
		
	Overall (O)	In HSIL and cancer (H)	
16	53.4	55.0	0.97
31	15.6	18.6	0.84
18	8.9	4.3	2.07
35	5.6	7.9	0.71
52	5.4	7.1	0.76
33	1.2	2.9	0.41
58	0.7	0.7	1.00
NI	0.7	3.6	0.19

NI, type not identified.

## Discussion

Studies on the prevalence of HPV cervical infection in Mexico are scarce [7,20-22] and information on HR-HPV prevalence is even scarcer [12,22] or deals only with HPV types 16 and 18 [23,24].

Since our aim was to determine the prevalence of the HR-HPV types causing cervical infections in the states of SLP and Gto, the study included women with cervical intraepithelial lesions (73.1% low grade, 16.7% high grade and 10.2% invasive carcinoma), expected to be HPV-positive. This prediction was fulfilled because nearly all women were shown to be infected with HPV, and HR types were identified in most of them.

To determine HPV infection and to identify the high-risk types involved we used the PCR-RFLP method based on amplification of the E6-250 HR-HPV product followed by digestion with endonucleases [19] and fragment sizing in high resolution agarose gels [25]. Obtaining cervical scrapings with the cytobrush allowed us to obtain more than enough amplifiable DNA in nearly all samples and PCR optimization assured efficient amplification with the primer sets employed.

**Figure 4:**
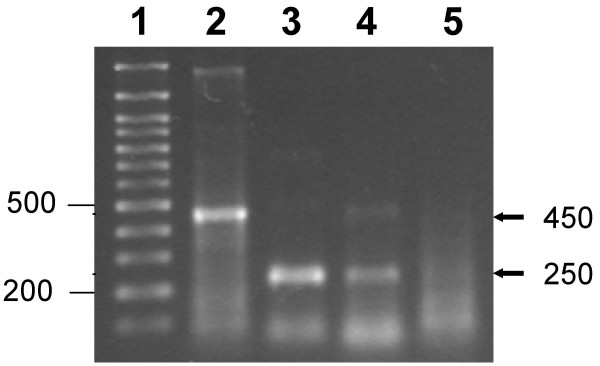
**Inhibition of L1-450 and E6-250 segment amplification in duplex PCR mixtures with primer sets MY 09/11 and pU 1M/2R**. Mixtures contained 50 ng DNA from Patient 1 as well as primer set MY 09/11, pU 1M/2R, or both. Arrows to the left indicate positions of the bands expected. Lane 1, 100 bp ladder. Lane 2, Uniplex 1 mixture (set MY 09/11). Lane 3, Uniplex mixture 2 (set pU 1M/2R). Lane 4, Duplex mixture (sets MY 09/11 and pU 1M/2R). Lane 5, Negative control (without DNA). Major bands expected in uniplex and duplex mixtures: ~450 bp with set MY 09/11 (Lanes 2 and 4) and ~250 bp with set pU 1M/2R (Lanes 3 and 4). Note the decrease in the intensity of bands in the duplex mixture (lane 4), especially ~450 bp.

The E6-250 HR-HPV product was amplified through nested PCR because this modality turned out to be at least twice as sensitive as direct PCR. The universal primer sets for the first ('direct') reaction amplified the E6-650 product [19,26] and the primer set used in the second ('nested') reaction amplified the E6-250 product [27]. The use of duplex PCR was discarded to amplify E6-250 and L1-450 due to the interference observed when simultaneous amplification of both products was attempted (Fig. 4). The scarce E6-negative samples were tested later with a universal primer set that amplifies L1-450 segments of both low risk- as well as HR-HPV types [28]. From these observations we concluded that to determine HR-HPV infection in women with dysplasia and neoplasia it is convenient first to identify the HPV-positive samples and then to check the amplificability of the negative ones.

Eight HR-HPV types (16, 18, 31, 33, 35, 45, 52 and 58) are responsible for 95% of the invasive CC cases in the world [14]. The PCR-RFLP used in this work identified all of them except type 45.

Considering all cervical samples included in this work, 98.8% contained amplifiable DNA, 97.8% had HPV infections which in 90.2% of the cases corresponded to HR-HPV types whose prevalence was higher in Gto (99.0%) than in SLP (82.6%) (Table 3).

In the 437 samples with amplifiable DNA included in this study, 528 cervical infections were demonstrated by HR-HPV types that were identified in 99.1% of the cases (Table 4). In this way we demonstrated that in our region circulate the seven HR-HPV types identified with the method used and their overall prevalence is, in descending order, 16 (53.4%) > 31 (15.6%) > 18 (8.9%) > 35 (5.6) > 52 (5.4%) > 33 (1.2%) > 58 (0.7%) = unidentified types (0.7%). From these results we recommend that for analysis of cervical samples with dysplasia it is convenient to identify first the types 16, 18 and 33 by restriction with *Ava *II and then the types 31, 35, 52 and 58 by restriction with *Rsa *I, *Ava *I, *Bgl *II and *Acc *I.

The prevalence of high and low risk HPV types found by us is similar to that already seen in Mexico and other regions of high CC incidence [20,21,29]. The predominant HR-HPV types in our region are 16, 31 and 18 which together represent 77.9% of all cervical infections. The four remaining types cause 12.9%, and unidentified types only 0.7% of the HR-HPV infections. Types 16 and 18 are slightly more prevalent in SLP, whereas type 31 is nearly two times more prevalent in Gto.

The overall prevalence of HPV type 16 in our region (53.4%) is almost identical to the world average (53%) calculated by Munoz (2000). In contrast, the regional prevalence of type 18 (8.9%) is substantially lower than the world average (15%) calculated by Munoz [21]. The prevalence of types 31 (15.6%) and 35 (5.6%) is higher than the average for Latin America [21], whereas that of type 33 (1.2%) is nearly 10 times lower than the average for Central America (11.8%) recently obtained by Clifford et al. [30].

Single and triple infections were more frequent in SLP, whereas double infections were more frequent in Gto. Single infections included all seven HR-HPV identifiable types where the three predominant ones were, in descending order, 16 > 18 > 31 with a cumulative frequency of 92.7%. The prevalence of these types in SLP was, in descending order, 16 > 18 > 31 with a cumulative frequency of 95.0%, whereas in Gto it was 16 > 31 > 18 with a cumulative frequency of 89.1%.

The typing method allowed us to identify a considerable proportion of infections by two viral types (overall prevalence of 25.9%, with 22.6% for SLP and 29.5% for Gto) and three viral types (overall prevalence of 2.7%, with 2.4% for SLP and 3.2% for Gto) (Table 4). Type 16 was found in all multiple infections.

Double infections from SLP were caused by the seven identifiable types but type 58 was absent in Gto. The overall predominant types in double infections were 16 > 31 > 35 > 18 = 52. Their prevalence differed in each state since the descending order in SLP was 16 > 31 > 18 > 35 > 52, whereas in Gto it was 16 > 31 > 35 > 52 > 18 (Table 6).

Types 33 and 58 were absent from triple infections in both states and the overall predominant types were 16 > 35 > 31 = 52 > 18. There were also differences in the prevalence by state since in SLP the descending order was 16 > 35 = 52 > 31 > 18 whereas in Gto it was 16 > 35 = 52 > 31 > 18 (Table 6).

Our algorithm for typing, designed to identify first the high-risk viral types and then the low risk ones, probably underestimates the prevalence of low risk types in samples where high risk types are demonstrated. However, it is worth noting that in low grade intraepithelial cervical lesions practically all genital HPV types are found, whereas in high grade lesions only high risk types are found [31].

The proportion of multiple infections found by us (28.6%) is over three times higher than the world average (8.1%) calculated by Molano et al. [32]. This feature is probably due to the fact that the women in our study are themselves a high risk group since they were included for having cervical dysplasia and cancer. This idea is reinforced by our finding that 10.6% of the double infections are due to the 16/18 type couple, whose prevalence was nearly six times higher than the world average (1.8%) estimated by Clifford et al. [30].

The five predominant HR-HPV types (16, 31, 18, 35 and 52) found in the 140 HSIL and invasive cancer lesions accounted for 92.9% of the cases and their frequency was similar to their overall prevalence (Table 9).

Infections by two or more HPV types are markers of persistent cervical disease, of multiple cervical lesions and of progression from low to high grade lesions [33]. There is scarce evidence on the interaction of HPV types to enhance CC pathogenesis and it is unknown if some HPV types promote or exclude infection by others. Careful follow up of multiple infections may help to pinpoint the relevance of the interactions of different HPV types in disease progression [34].

HPV16, the most frequent type in single and multiple infections in SLP and Gto, just as in the rest of the world [14,21] appears to be the most persistent type [32,35,36] increasing the risk of developing severe preneoplastic lesions [37].

Our work confirms the usefulness of the PCR-RFLP method of Fujinaga et al. [19,26,27] for diagnosis and typing cervical infections by HR-HPV, whose sensitivity was at least doubled by amplifying the E6-250 product through nested rather than direct PCR and whose typing precision was improved by using high resolution electrophoresis. In this way we could identify the HR-HPV types circulating in our region and their distribution in the two neighboring states analyzed, which reflect the endemic behavior of the various HPV types as well as local environmental or genetic conditions of the hosts [38].

Besides being of use to carry out molecular epidemiology studies and to predict the individual risk of each patient, HR-HPV typing is essential to plan the administration of prophylactic vaccines directed against one or more specific HPV types, which is questionable in populations where prevalence of the circulating types is unknown [31,39,40]. Since in our region HPV type 16 has the highest overall prevalence (53.4%), type 31 is second (15.6%) and type 18 is third (8.9%), the vaccine against HR-HPV types 16 and 18 [41] whose license was granted first in Mexico and the United States [42,43] would be expected to cover at least these three types [44] and would have a 78% efficacy.

## Conclusion

Enough DNA is obtained from cervical scrapings of women with dysplasia in order to detect high risk-HPV by PCR amplification followed by typing through restriction fragment length polymorphism analysis.

Sensitivity of HR-HPV detection is nearly doubled by nested rather than direct PCR amplification.

Ninety-two percent of the 437 Mexican women with cervical dysplasia and cancer included in this study were infected by HR-HPV, with a prevalence 1.14 times higher in the state of Guanajuato than in San Luis Potosí.

All seven HR-HPV types identifiable with the method used (16, 18, 31, 33, 35, 52, 58) circulate in SLP and Gto and were diagnosed in nearly all (99.3%) the cases.

Seventy-one percent of the infections were caused by one HR-HPV type, 25.9% by two types and 2.7% by three types.

The overall frequency of cervical lesions was LSIL (73.1%) > HSIL (16.7%) > invasive cancer (10.2%) and the ratio of single to multiple infections was inversely proportional to the severity of the lesions: 2.46 for LSIL, 2.37 for HSIL and 2.15 for invasive cancer.

The frequency of HR-HPV types found in HSIL and invasive cancer was 16 (55.0%) > 31 (18.6%) > 35 (7.9%) > 52 (7.1%) > 18 (4.3%) > unidentified types (3.6%) > 33 (2.9%) > 58 (0.7%).

Since the three predominant HR-HPV types found (16, 31 and 18) cause 77.9% of the HR-HPV infections associated to cervical dysplasia in this region, and immunization against type 16 prevents type 31 infections, in this region the efficacy of the prophylactic vaccine against types 16 and 18 would be close to 80%.

## Methods

### Source of women

The 442 women included in this study were selected for having precancerous or cancerous lesions of the cervical epithelium confirmed by cytologic analysis. Two hundred and eighteen resided in the city of San Luis Potosí, capital of the state of San Luis Potosí (SLP), and 192 in the cities of León, Celaya and Irapuato from the state of Guanajuato (Gto), Mexico. Cervical scrapings from SLP were obtained at the Dysplasia Clinic of the SLP Health Services, the School of Nursing of Universidad Autónoma de San Luis Potosí, and from private patients. Samples from Gto were obtained at the Dysplasia Clinics of the cities of Irapuato, Celaya and León and sent to our laboratory by the Guanajuato Health Secretariat. The study was authorized by the Health Secretariats of SLP and Gto and performed with the informed consent of all participating women.

### Cervical scrapings and DNA extraction

Each scraping was taken with an endocervical brush ('cytobrush') that was immediately inserted in a 5 ml polypropylene tube (Nalge Nunc, Rochester, NY) containing 1 ml phosphate buffered saline supplemented with sterile disodium ethtylene-diamino-tetraacetate (PBS-EDTA: 137 mM NaCl, 2.7 mM KCl, 10 mM Na_2_HPO_4_, 2 mM KH_2_HPO_2_, 25 mM disodium EDTA, pH 7.4). Once detached from the cytobrush and suspended in the PBS-EDTA vehicle, each sample was fixed by addition of 1.5 ml 96% ethanol and processed to extract DNA on the same day or up to 30 days after being kept at room temperature. Reagents were purchased from J.T. Baker (Xalostoc, México) unless other source is specified.

To extract the DNA each fixed sample was mixed by vortexing and 1 ml transfered to a 1.5 ml tube and spun in a Hettich Mikro 20 microcentrifuge (Cologne, Germany) for 5 min at 13,000 rpm (16,250×*g*). The supernatant was discarded by decantation and to each pellet were added 500 μl of Tris-EDTA-saline (TES: 10 mM Tris-HCl; 2 mM disodium EDTA, 0.4 M NaCl, pH 8.0 at 25°C), 50 μl of 10% sodium dodecyl sulphate and 20 μl of proteinase K (20 mg/ml). Mixtures were incubated at 55°C for 3 h, at the end of which 150 μl of 5 M NaCl were added and centrifuged again for 15 min. Each supernatant was aspirated and transferred to a tube to which 577 μl cold isopropanol were immediately added and then left stand for 10 min at 4°C to precipitate the nucleic acids. The tubes were centrifuged again for 10 min and supernatants discarded by decantation. Each pellet was washed by vortexing with 1 ml of 70% cold ethanol and centrifuged for 10 min at 10,000 rpm (9,615×*g*) and room temperature. Supernatants were discarded by aspiration and the pellets dried out by inverting the tubes for 15 min on a paper towel. Each pellet was dissolved with 50 μl TE (10 mM Tris-HCl, 1 mM disodium EDTA, pH 8.0 at 25°C).

The quality of extracted DNA was verified by electrophoretic analysis in 1% agarose gels with TAE (40 mM Tris-acetate, 1 mM disodium EDTA, pH 8.2 at 25°C). Two-μl from each sample were applied to gels which were run at 60 V for 90 min. λ-phage? DNA digested with *Hind *III (Sigma-Aldrich, Mexico) was used as marker. After staining for 20 min with ethidium bromide (1 μg/ml) gels were illuminated with ultraviolet light and their fluorescence recorded with the Bio-Rad ChemiDoc EQ (Hercules, CA) photo documenter.

DNA was quantitated by fluorometry with the PicoGreen dsDNA Quantitation kit (Molecular Probes; Eugene, OR) by interpolation in a standard curve containing up to 50 ng of λ-phage DNA. To each well of a black FIA 96 well plate (Greiner Bio-One, Frickenhausen, Germany) 198 μl of the assay solution (PicoGreen diluted 1:400 in TE) and 2 μl of standard DNA or problem samples were added, and their fluorescence determined using a 485 nm excitation filter and a 535 emission filter in the GENios Pro fluorometer (Tecan Systems, San Jose, CA) with the Magellan 4 software.

Since amplification of the β-globin gene required = 25 ng DNA per PCR mixture, 100 ng was the minimum amount of DNA required from each cervical scraping in order to prepare at least four PCR mixtures.

The range of pure DNA obtained from the first 370 cervical scrapings was 53–16,590 ng. The amount of purified DNA was sufficient (i.e., = 100 ng) in all samples except five of the initial ones.

### PCR conditions

The oligonucleotide primer sets and the sizes expected for the HPV amplification products are depicted in Table 10. To optimize PCR with each primer set variable DNA and MgCl_2 _concentrations and annealing temperatures were tested. The β-globin primer set was added to PCR mixtures containing double-serial dilutions of DNA from a HPV-positive sample (range: 0.78–50 ng). To optimize conditions for HPV primer sets, 1–5 mM MgCl_2 _concentrations and annealing temperatures from 55°C to 61.4°C were tested.

**Table 10:** Oligonucleotides used

		Oligonucleotides		Gene amplified	Amplicon size
		
Set	Name	Sequence (5' → 3')	Position^a^		
1^b^	LCRS	AAGGGAGTAACCGAAAACGGT	26	E6	~650 bp
	E7AS	TCATCCTCCTCCTCTGAG	671		
2^b^	pU1M	TGTCAAAAACCGTTGTGTCC	419	E6	~250 bp
	pU2R	GAGCTGTCGCTTAATTGCTC	656		
3^b^	MY09	CGTCCMARRGGAWACTGATC	6584	L1	~450 bp
	MY11	GCMCAGGGWCATAAYAATGG	7035		
4^c^	PC04	CAACTTCATCCACGTTCAACC	---	β-globin	~260 bp
	GH20	GAAGAGCCAAGGACAGGTAC	---		

^a ^Nucleotide of the HPV16 genome at which the 5' end of each primer is bound.

^b ^Fujinaga et al. [19].

^c ^Sotlar et al. [46].

To maximize HPV detection through PCR amplification, three variables were optimized: DNA content, annealing temperature and magnesium concentration.

With the PC04/GH20 primer set for β-globin the minimum amount of template DNA required in PCR mixtures was determined with double serial dilutions leading to a maximum of 50 ng DNA per mixture. Fluorescence of the E6-250 product increased with DNA content up to 25 ng, which was the amount selected to amplify HPV DNA from each sample. Annealing temperature in the range of 55°C to 62°C was assessed with each primer set. As major products attained maximum fluorescence at 57°C this annealing temperature was used with all primer sets for the rest of the work. MgCl_2 _concentrations in the 1–5 mM range were tested. Maximum efficiency was attained at 2.5 mM, concentration chosen for the rest of the work.

The primer set LCRS/E7AS amplifies the E6-650 segment and the set pU 1M/2R amplifies the 'nested' E6-250 segment included in the first one. In the HPV16 genome, the set LCRS/E7AS spans nucleotides 26–671 (645 bp) and the set pU 1M/2R spans nt 419–656 (237 bp). The set MY 09/11 amplifies the L1-450 segment which spans nt 6584–7035 (451 bp). To explore the feasibility of using duplex PCR to simultaneously amplify E6-250 with primer set pU 1M/2R and L1-450 with primer set MY 09/11, we tested both sets at 0.6 μM concentration. However, our finding that in duplex mixtures the L1-450 band was nearly lost and the E6-250 band was less intense than in uniplex mixtures (Fig. 4) led us to discard the use of duplex mixtures.

### Detection of the HPV DNA amplification products

LCR/E7 ~650 bp (E6-650), E6/E7 ~250 bp (E6-250) and L1 ~450 bp (L1-450) products were amplified with primer sets pU 1M/2R, LCRS/E7AS and MY 09/11, respectively (Table 7). Each 50 μl PCR mixture was prepared in 200 μl Axygen tubes (Union City, CA). PCR mixtures contained 2.5 mM MgCl_2_, 0.4 mM of each dNTP, 0.6 μM of each oligonucleotide, 1.5 units of recombinant Taq DNA polymerase and 25 ng DNA, 20 mM Tris-HCl, 50 mM KCl, pH 8.4 at 25°C; oligonucleotides were synthesized by Accesolab (Mexico) and other components were purchased from Invitrogen (Mexico).

PCR mixtures were incubated 40 cycles in a Techne Touchgene Gradient thermocycler (Staffordshire, England). Before starting they were incubated at 94°C for 4 min. Each cycle consisted of 1 min periods at 94°C (denaturation), 57°C (annealing) and 72°C (extension), followed by 10 min final extension at 72°C. Each run included a positive control with 25 ng of HeLa cell DNA known to contain 10–50 copies of the HPV type 18 genome per cell [45], and a negative control with water instead of DNA.

PCR products (10 μl from each mixture) were subjected to electrophoresis in 1.5% agarose gels with 5 mM sodium borate, pH 8.5 [25] that were run at 120 V for 90 min.

### High-risk HPV typing

To identify HR-HPV types the method of Fujinaga et al. [19] was used. The size of the two E6-250 restriction fragments generated with endonucleases for each of the seven HR-HPV types that can be identified with this method are shown in Table 2.

*Ava *I, *Ava *II and *Bgl *II endonucleases and their buffers were purchased from Biolabs (Beverly, MA), whereas *Rsa *I and *Acc *I and their buffers were obtained from Invitrogen (Carlsbad, CA). Digestion mixtures containing 8 μl of PCR amplification mixture, 1.5 μl endonuclease solution (10 U/μl), 2 μl buffer and 8.5 μl water were incubated 3 h at 37°C. The restriction fragments present in 10 μl from each digestion mixture were analyzed by electrophoresis in 2% agarose gels with 5 mM sodium borate, pH 8.5 (Brody and Kern 2004) that were run at 120 V for 90 min.

## Competing interests

The author(s) declare that they have no competing interests.

## Authors' contributions

LAMC performed most of the molecular studies. MSG suggested and set up the PCR-RFLP method. RLR conceived and designed the study, obtained the funds to carry it out and drafted the manuscript. All authors read and approved the final manuscript.
